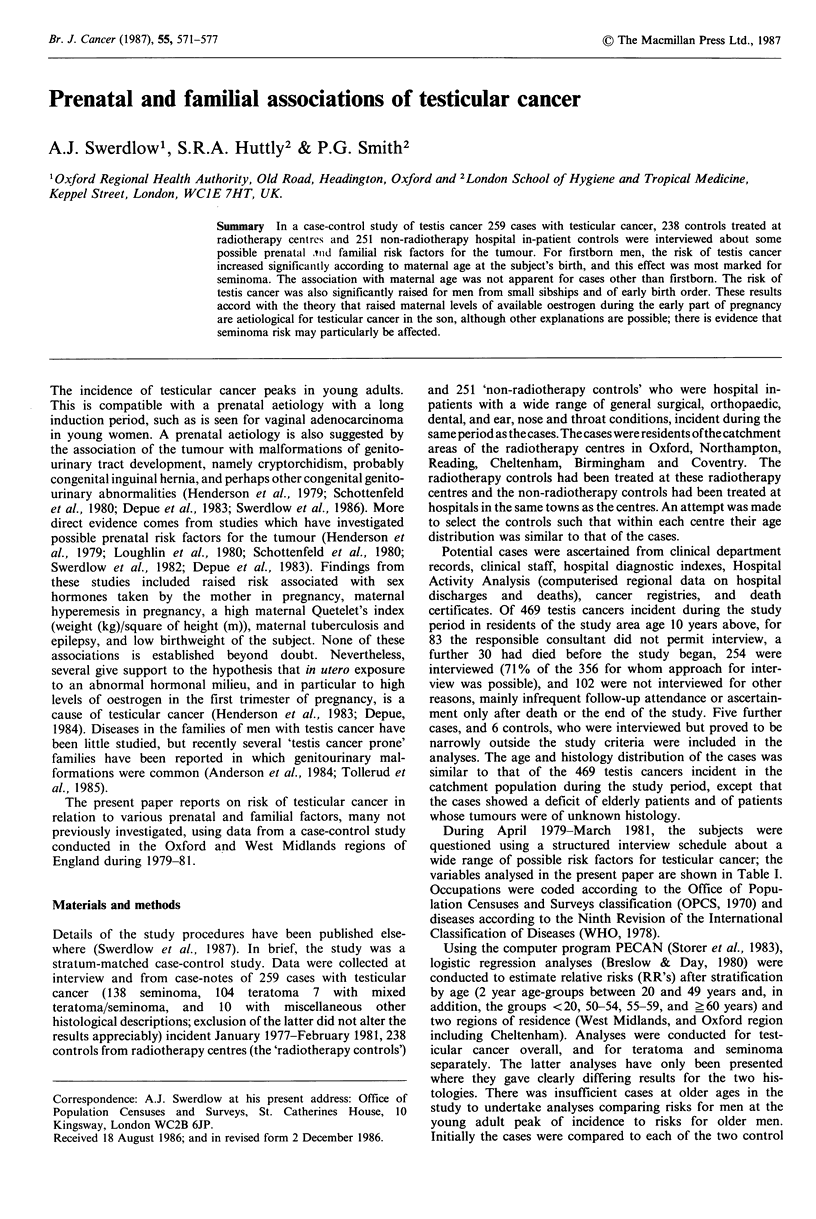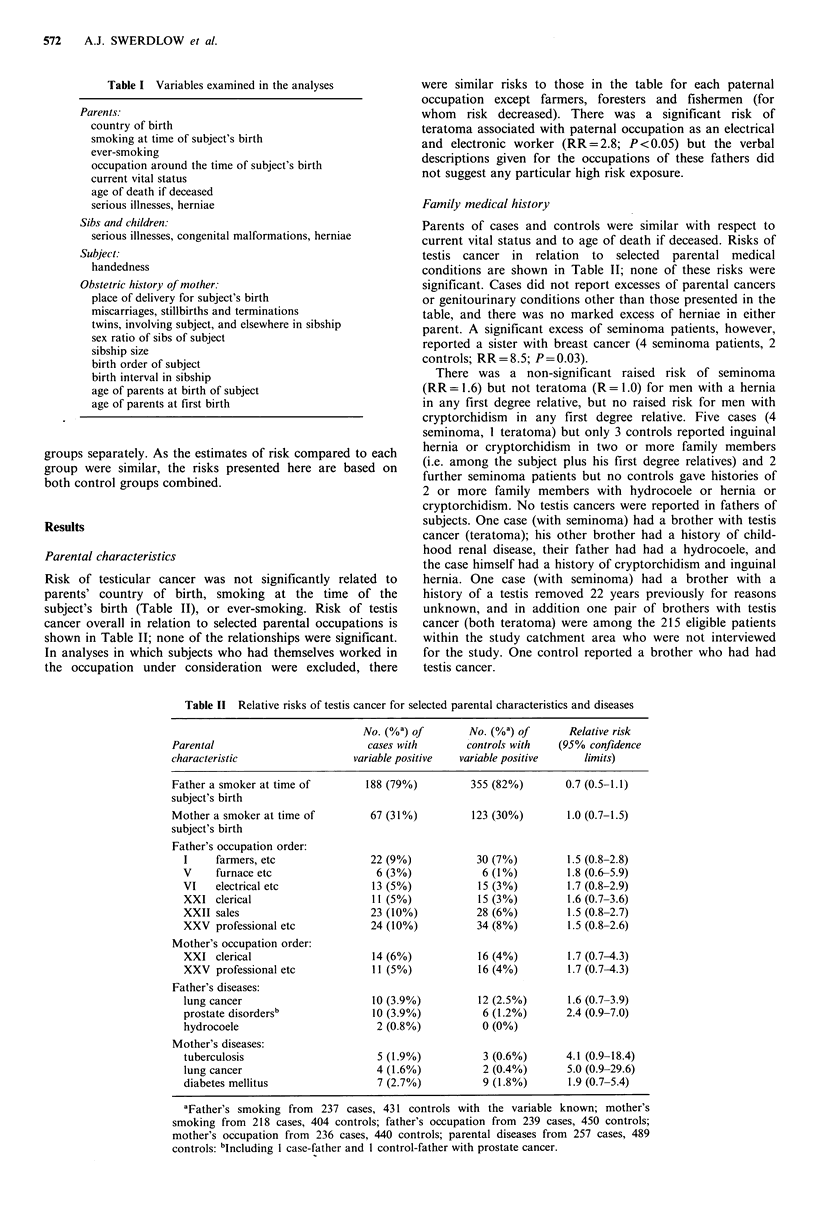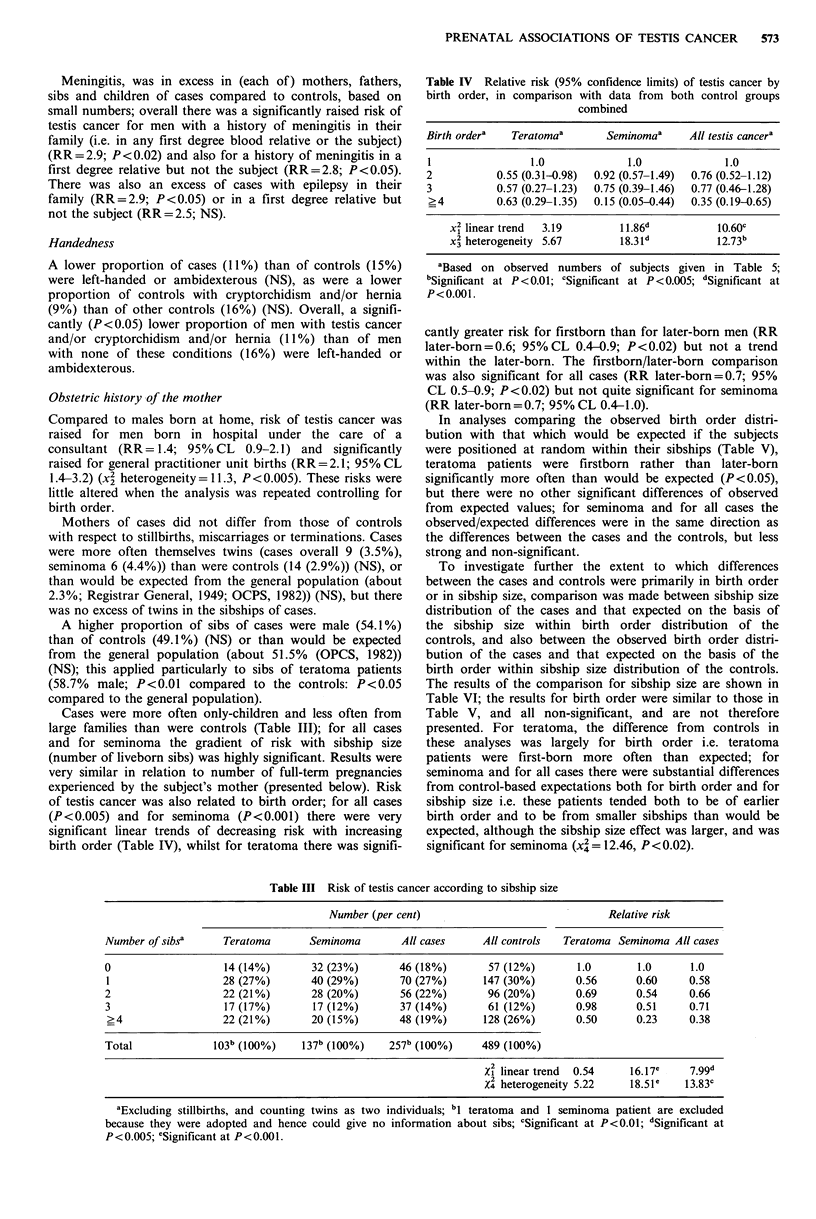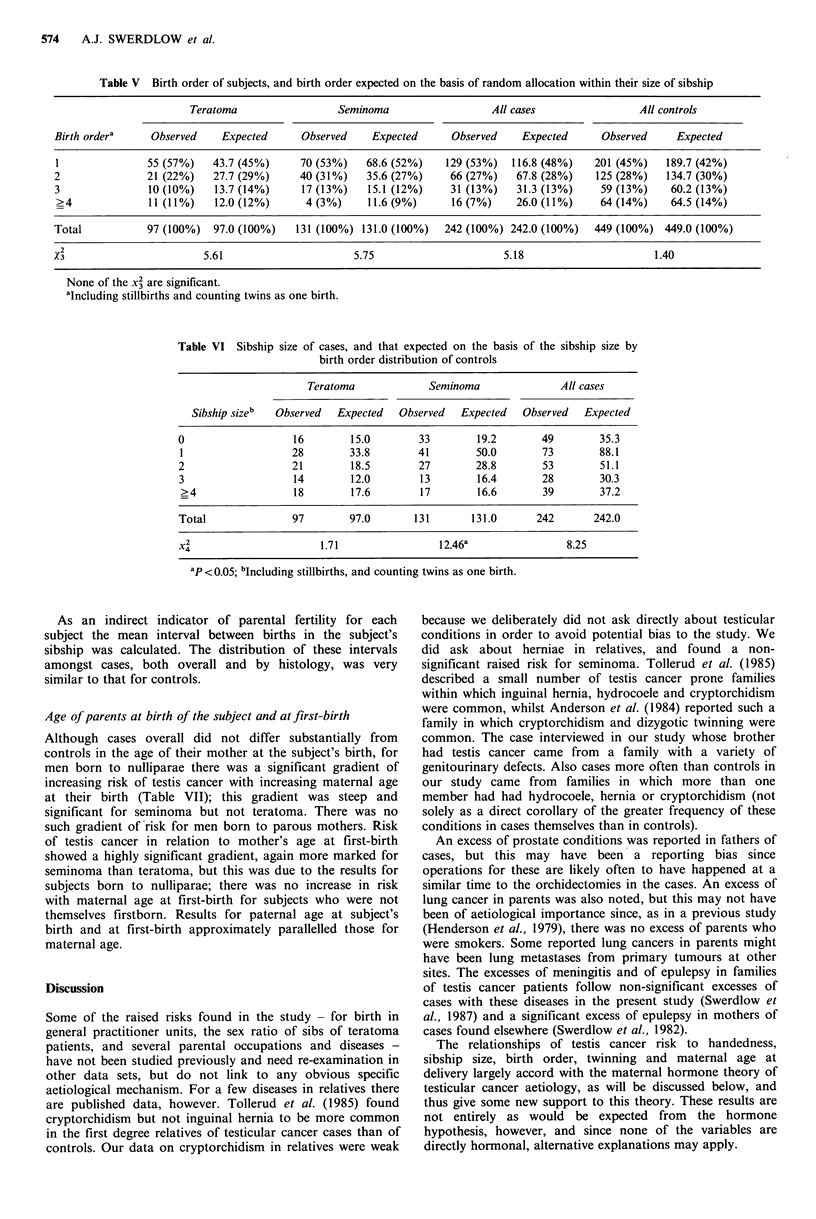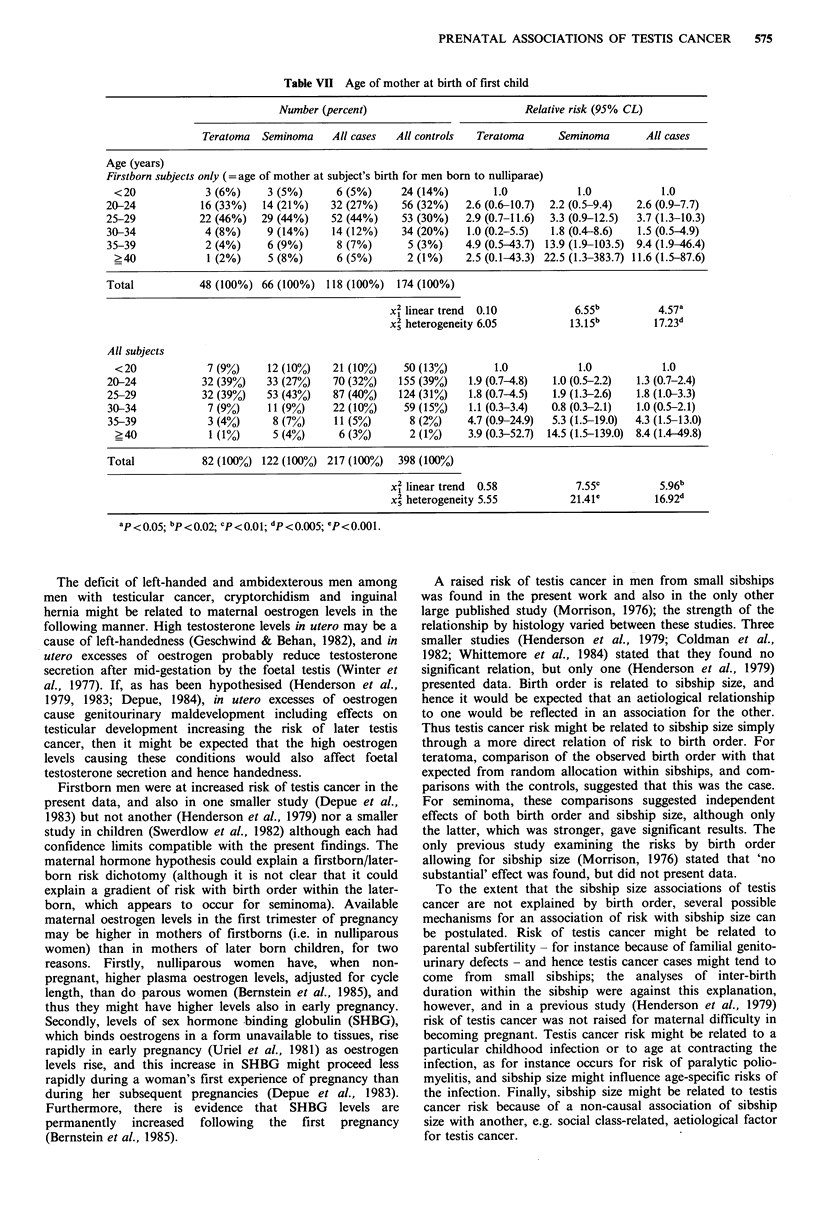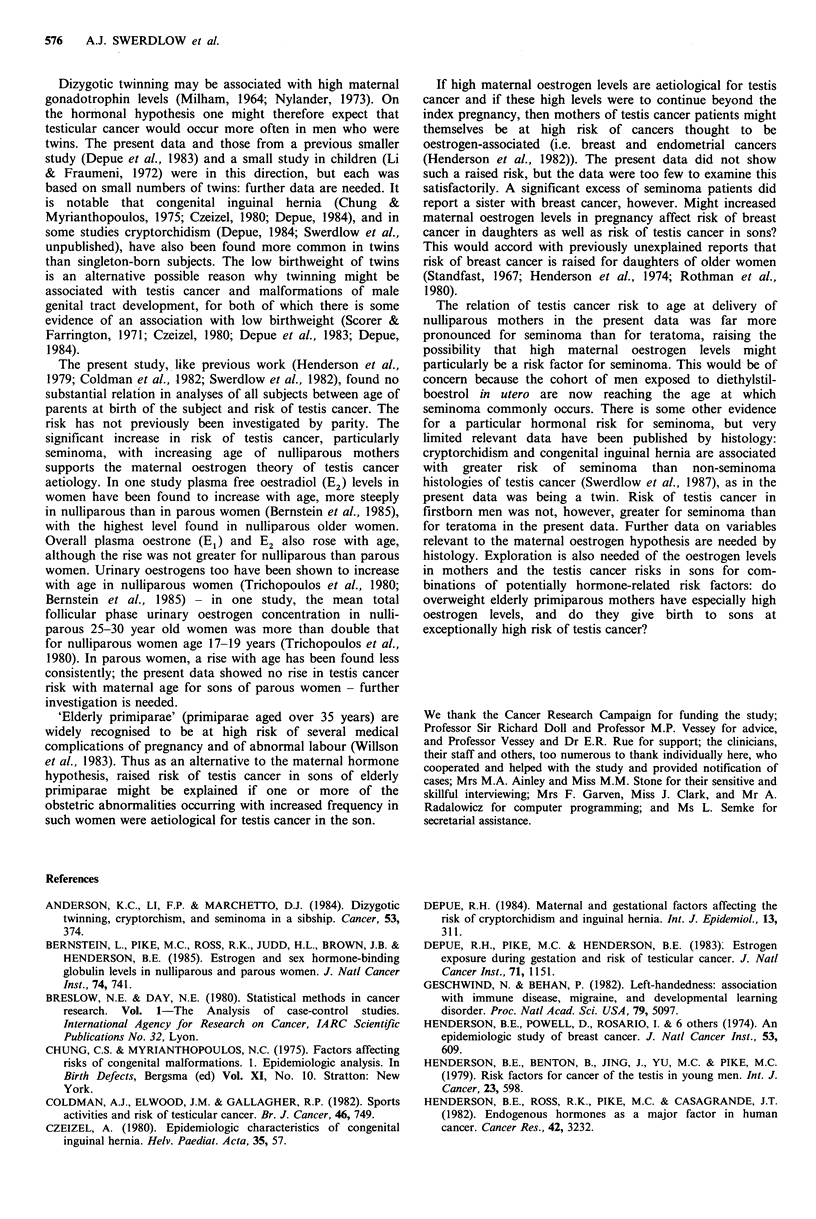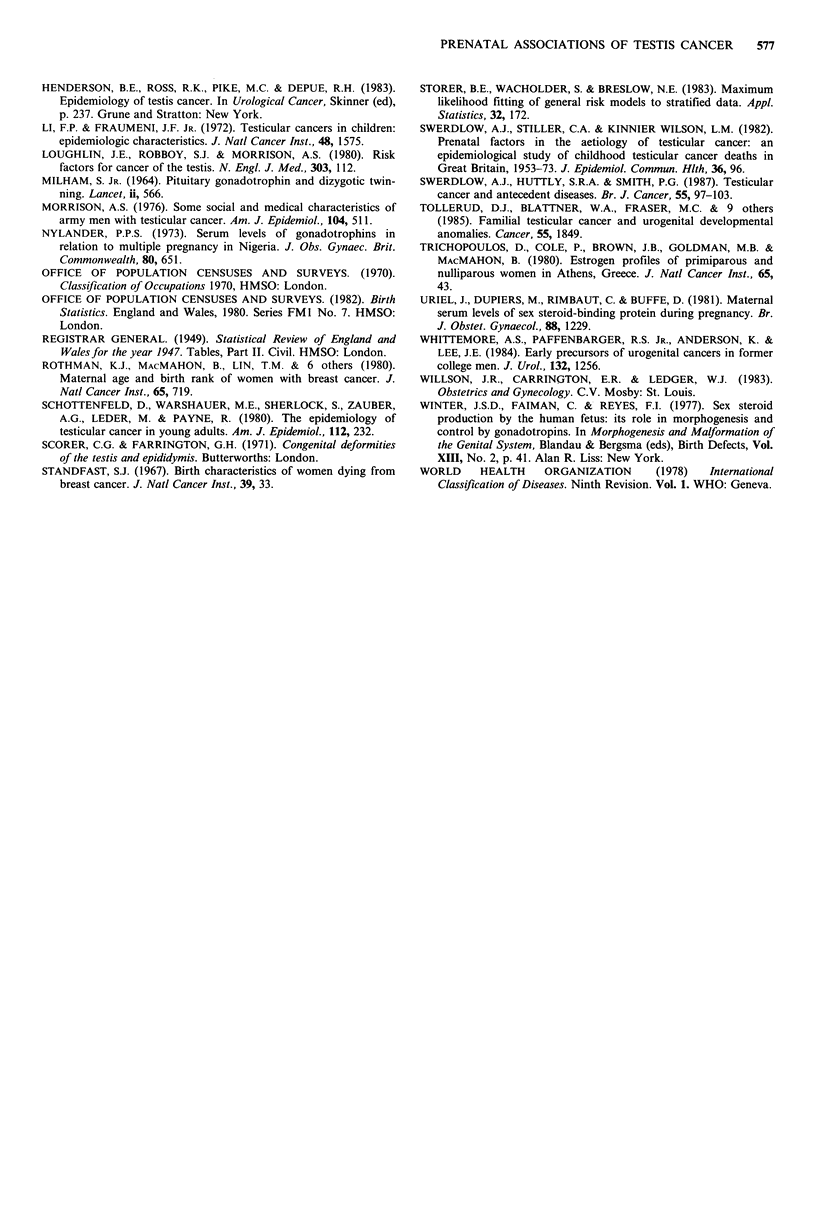# Prenatal and familial associations of testicular cancer.

**DOI:** 10.1038/bjc.1987.116

**Published:** 1987-05

**Authors:** A. J. Swerdlow, S. R. Huttly, P. G. Smith

## Abstract

In a case-control study of testis cancer 259 cases with testicular cancer, 238 controls treated at radiotherapy centres and 251 non-radiotherapy hospital in-patient controls were interviewed about some possible prenatal and familial risk factors for the tumour. For firstborn men, the risk of testis cancer increased significantly according to maternal age at the subject's birth, and this effect was most marked for seminoma. The association with maternal age was not apparent for cases other than firstborn. The risk of testis cancer was also significantly raised for men from small sibships and of early birth order. These results accord with the theory that raised maternal levels of available oestrogen during the early part of pregnancy are aetiological for testicular cancer in the son, although other explanations are possible; there is evidence that seminoma risk may particularly be affected.


Br. J. Cancer (1987), 55, 571–577 © The Macmillan Press Ltd., 1987

### Prenatal and familial associations of testicular cancer

A.J. Swerdlow[1], S.R.A. Huttly[2] & P.G. Smith[2]

[1]Oxford Regional Health Authority, Old Road, Headington, Oxford and [2]London School of Hygiene and Tropical Medicine, Keppel Street, London, WC1E 7HT, UK.

**Summary** In a case-control study of testis cancer 259 cases with testicular cancer, 238 controls treated at radiotherapy centres and 251 non-radiotherapy hospital in-patient controls were interviewed about some possible prenatal and familial risk factors for the tumour. For firstborn men, the risk of testis cancer increased significantly according to maternal age at the subject's birth, and this effect was most marked for seminoma. The association with maternal age was not apparent for cases other than firstborn. The risk of testis cancer was also significantly raised for men from small sibships and of early birth order. These results accord with the theory that raised maternal levels of available oestrogen during the early part of pregnancy are aetiological for testicular cancer in the son, although other explanations are possible; there is evidence that seminoma risk may particularly be affected.

The incidence of testicular cancer peaks in young adults. This is compatible with a prenatal aetiology with a long induction period, such as is seen for vaginal adenocarcinoma in young women. A prenatal aetiology is also suggested by the association of the tumour with malformations of genito-urinary tract development, namely cryptorchidism, probably congenital inguinal hernia, and perhaps other congenital genito-urinary abnormalities (Henderson *et al.*, 1979; Schottenfeld *et al.*, 1980; Depue *et al.*, 1983; Swerdlow *et al.*, 1986). More direct evidence comes from studies which have investigated possible prenatal risk factors for the tumour (Henderson *et al.*, 1979; Loughlin *et al.*, 1980; Schottenfeld *et al.*, 1980; Swerdlow *et al.*, 1982; Depue *et al.*, 1983). Findings from these studies included raised risk associated with sex hormones taken by the mother in pregnancy, maternal hyperemesis in pregnancy, a high maternal Quetelet's index (weight (kg)/square of height (m)), maternal tuberculosis and epilepsy, and low birthweight of the subject. None of these associations is established beyond doubt. Nevertheless, several give support to the hypothesis that *in utero* exposure to an abnormal hormonal milieu, and in particular to high levels of oestrogen in the first trimester of pregnancy, is a cause of testicular cancer (Henderson *et al.*, 1983; Depue, 1984). Diseases in the families of men with testis cancer have been little studied, but recently several 'testis cancer prone' families have been reported in which genitourinary malformations were common (Anderson *et al.*, 1984; Tollerud *et al.*, 1985).

The present paper reports on risk of testicular cancer in relation to various prenatal and familial factors, many not previously investigated, using data from a case-control study conducted in the Oxford and West Midlands regions of England during 1979–81.

### Materials and methods

Details of the study procedures have been published elsewhere (Swerdlow *et al.*, 1987). In brief, the study was a stratum-matched case-control study. Data were collected at interview and from case-notes of 259 cases with testicular cancer (138 seminoma, 104 teratoma 7 with mixed teratoma/seminoma, and 10 with miscellaneous other histological descriptions; exclusion of the latter did not alter the results appreciably) incident January 1977–February 1981, 238 controls from radiotherapy centres (the 'radiotherapy controls') and 251 'non-radiotherapy controls' who were hospital in-patients with a wide range of general surgical, orthopaedic, dental, and ear, nose and throat conditions, incident during the same period as the cases. The cases were residents of the catchment areas of the radiotherapy centres in Oxford, Northampton, Reading, Cheltenham, Birmingham and Coventry. The radiotherapy controls had been treated at these radiotherapy centres and the non-radiotherapy controls had been treated at hospitals in the same towns as the centres. An attempt was made to select the controls such that within each centre their age distribution was similar to that of the cases.

Potential cases were ascertained from clinical department records, clinical staff, hospital diagnostic indexes, Hospital Activity Analysis (computerised regional data on hospital discharges and deaths), cancer registries, and death certificates. Of 469 testis cancers incident during the study period in residents of the study area age 10 years above, for 83 the responsible consultant did not permit interview, a further 30 had died before the study began, 254 were interviewed (71% of the 356 for whom approach for interview was possible), and 102 were not interviewed for other reasons, mainly infrequent follow-up attendance or ascertainment only after death or the end of the study. Five further cases, and 6 controls, who were interviewed but proved to be narrowly outside the study criteria were included in the analyses. The age and histology distribution of the cases was similar to that of the 469 testis cancers incident in the catchment population during the study period, except that the cases showed a deficit of elderly patients and of patients whose tumours were of unknown histology.

During April 1979–March 1981, the subjects were questioned using a structured interview schedule about a wide range of possible risk factors for testicular cancer; the variables analysed in the present paper are shown in Table I. Occupations were coded according to the Office of Population Censuses and Surveys classification (OPCS, 1970) and diseases according to the Ninth Revision of the International Classification of Diseases (WHO, 1978).

Using the computer program PECAN (Storer *et al.*, 1983), logistic regression analyses (Breslow & Day, 1980) were conducted to estimate relative risks (RR's) after stratification by age (2 year age-groups between 20 and 49 years and, in addition, the groups <20, 50–54, 55–59, and ≥60 years) and two regions of residence (West Midlands, and Oxford region including Cheltenham). Analyses were conducted for testicular cancer overall, and for teratoma and seminoma separately. The latter analyses have only been presented where they gave clearly differing results for the two histologies. There was insufficient cases at older ages in the study to undertake analyses comparing risks for men at the young adult peak of incidence to risks for older men. Initially the cases were compared to each of the two control

Correspondence: A.J. Swerdlow at his present address: Office of Population Censuses and Surveys, St. Catherines House, 10 Kingsway, London WC2B 6JP.
Received 18 August 1986; and in revised form 2 December 1986.

**Table I** Variables examined in the analyses

| |
|---|
| *Parents:* |
| country of birth |
| smoking at time of subject's birth |
| ever-smoking |
| occupation around the time of subject's birth |
| current vital status |
| age of death if deceased |
| serious illnesses, herniae |
| *Sibs and children:* |
| serious illnesses, congenital malformations, herniae |
| *Subject:* |
| handedness |
| *Obstetric history of mother:* |
| place of delivery for subject's birth |
| miscarriages, stillbirths and terminations |
| twins, involving subject, and elsewhere in sibship |
| sex ratio of sibs of subject |
| sibship size |
| birth order of subject |
| birth interval in sibship |
| age of parents at birth of subject |
| age of parents at first birth |

groups separately. As the estimates of risk compared to each group were similar, the risks presented here are based on both control groups combined.

### Results

### *Parental characteristics*

Risk of testicular cancer was not significantly related to parents' country of birth, smoking at the time of the subject's birth (Table II), or ever-smoking. Risk of testis cancer overall in relation to selected parental occupations is shown in Table II; none of the relationships were significant. In analyses in which subjects who had themselves worked in the occupation under consideration were excluded, there were similar risks to those in the table for each paternal occupation except farmers, foresters and fishermen (for whom risk decreased). There was a significant risk of teratoma associated with paternal occupation as an electrical and electronic worker (RR = 2.8; $P<0.05$) but the verbal descriptions given for the occupations of these fathers did not suggest any particular high risk exposure.

### *Family medical history*

Parents of cases and controls were similar with respect to current vital status and to age of death if deceased. Risks of testis cancer in relation to selected parental medical conditions are shown in Table II; none of these risks were significant. Cases did not report excesses of parental cancers or genitourinary conditions other than those presented in the table, and there was no marked excess of herniae in either parent. A significant excess of seminoma patients, however, reported a sister with breast cancer (4 seminoma patients, 2 controls; RR = 8.5; $P = 0.03$).

There was a non-significant raised risk of seminoma (RR = 1.6) but not teratoma (R = 1.0) for men with a hernia in any first degree relative, but no raised risk for men with cryptorchidism in any first degree relative. Five cases (4 seminoma, 1 teratoma) but only 3 controls reported inguinal hernia or cryptorchidism in two or more family members (i.e. among the subject plus his first degree relatives) and 2 further seminoma patients but no controls gave histories of 2 or more family members with hydrocoele or hernia or cryptorchidism. No testis cancers were reported in fathers of subjects. One case (with seminoma) had a brother with testis cancer (teratoma); his other brother had a history of child-hood renal disease, their father had had a hydrocoele, and the case himself had a history of cryptorchidism and inguinal hernia. One case (with seminoma) had a brother with a history of a testis removed 22 years previously for reasons unknown, and in addition one pair of brothers with testis cancer (both teratoma) were among the 215 eligible patients within the study catchment area who were not interviewed for the study. One control reported a brother who had had testis cancer.

**Table II** Relative risks of testis cancer for selected parental characteristics and diseases

| *Parental characteristic* | *No. (%[a]) of cases with variable positive* | *No. (%[a]) of controls with variable positive* | *Relative risk (95% confidence limits)* |
|---|---|---|---|
| Father a smoker at time of subject's birth | 188 (79%) | 355 (82%) | 0.7 (0.5–1.1) |
| Mother a smoker at time of subject's birth | 67 (31%) | 123 (30%) | 1.0 (0.7–1.5) |
| Father's occupation order: | | | |
| I farmers, etc | 22 (9%) | 30 (7%) | 1.5 (0.8–2.8) |
| V furnace etc | 6 (3%) | 6 (1%) | 1.8 (0.6–5.9) |
| VI electrical etc | 13 (5%) | 15 (3%) | 1.7 (0.8–2.9) |
| XXI clerical | 11 (5%) | 15 (3%) | 1.6 (0.7–3.6) |
| XXII sales | 23 (10%) | 28 (6%) | 1.5 (0.8–2.7) |
| XXV professional etc | 24 (10%) | 34 (8%) | 1.5 (0.8–2.6) |
| Mother's occupation order: | | | |
| XXI clerical | 14 (6%) | 16 (4%) | 1.7 (0.7–4.3) |
| XXV professional etc | 11 (5%) | 16 (4%) | 1.7 (0.7–4.3) |
| Father's diseases: | | | |
| lung cancer | 10 (3.9%) | 12 (2.5%) | 1.6 (0.7–3.9) |
| prostate disorders[b] | 10 (3.9%) | 6 (1.2%) | 2.4 (0.9–7.0) |
| hydrocoele | 2 (0.8%) | 0 (0%) | |
| Mother's diseases: | | | |
| tuberculosis | 5 (1.9%) | 3 (0.6%) | 4.1 (0.9–18.4) |
| lung cancer | 4 (1.6%) | 2 (0.4%) | 5.0 (0.9–29.6) |
| diabetes mellitus | 7 (2.7%) | 9 (1.8%) | 1.9 (0.7–5.4) |

[a]Father's smoking from 237 cases, 431 controls with the variable known; mother's smoking from 218 cases, 404 controls; father's occupation from 239 cases, 450 controls; mother's occupation from 236 cases, 440 controls; parental diseases from 257 cases, 489 controls: [b]Including 1 case-father and 1 control-father with prostate cancer.

Meningitis, was in excess in (each of) mothers, fathers, sibs and children of cases compared to controls, based on small numbers; overall there was a significantly raised risk of testis cancer for men with a history of meningitis in their family (i.e. in any first degree blood relative or the subject) (RR = 2.9; $P<0.02$) and also for a history of meningitis in a first degree relative but not the subject (RR = 2.8; $P<0.05$). There was also an excess of cases with epilepsy in their family (RR = 2.9; $P<0.05$) or in a first degree relative but not the subject (RR = 2.5; NS).

*Handedness*

A lower proportion of cases (11%) than of controls (15%) were left-handed or ambidexterous (NS), as were a lower proportion of controls with cryptorchidism and/or hernia (9%) than of other controls (16%) (NS). Overall, a significantly ($P<0.05$) lower proportion of men with testis cancer and/or cryptorchidism and/or hernia (11%) than of men with none of these conditions (16%) were left-handed or ambidexterous.

*Obstetric history of the mother*

Compared to males born at home, risk of testis cancer was raised for men born in hospital under the care of a consultant (RR = 1.4; 95% CL 0.9–2.1) and significantly raised for general practitioner unit births (RR = 2.1; 95% CL 1.4–3.2) ($x_2^2$ heterogeneity = 11.3, $P<0.005$). These risks were little altered when the analysis was repeated controlling for birth order.

Mothers of cases did not differ from those of controls with respect to stillbirths, miscarriages or terminations. Cases were more often themselves twins (cases overall 9 (3.5%), seminoma 6 (4.4%)) than were controls (14 (2.9%)) (NS), or than would be expected from the general population (about 2.3%; Registrar General, 1949; OCPS, 1982)) (NS), but there was no excess of twins in the sibships of cases.

A higher proportion of sibs of cases were male (54.1%) than of controls (49.1%) (NS) or than would be expected from the general population (about 51.5% (OPCS, 1982)) (NS); this applied particularly to sibs of teratoma patients (58.7% male; $P<0.01$ compared to the controls: $P<0.05$ compared to the general population).

Cases were more often only-children and less often from large families than were controls (Table III); for all cases and for seminoma the gradient of risk with sibship size (number of liveborn sibs) was highly significant. Results were very similar in relation to number of full-term pregnancies experienced by the subject's mother (presented below). Risk of testis cancer was also related to birth order; for all cases ($P<0.005$) and for seminoma ($P<0.001$) there were very significant linear trends of decreasing risk with increasing birth order (Table IV), whilst for teratoma there was significantly greater risk for firstborn than for later-born men (RR later-born = 0.6; 95% CL 0.4–0.9; $P<0.02$) but not a trend within the later-born. The firstborn/later-born comparison was also significant for all cases (RR later-born = 0.7; 95% CL 0.5–0.9; $P<0.02$) but not quite significant for seminoma (RR later-born = 0.7; 95% CL 0.4–1.0).

**Table IV** Relative risk (95% confidence limits) of testis cancer by birth order, in comparison with data from both control groups combined

| *Birth order*[a] | *Teratoma*[a] | *Seminoma*[a] | *All testis cancer*[a] |
|---|---|---|---|
| 1 | 1.0 | 1.0 | 1.0 |
| 2 | 0.55 (0.31–0.98) | 0.92 (0.57–1.49) | 0.76 (0.52–1.12) |
| 3 | 0.57 (0.27–1.23) | 0.75 (0.39–1.46) | 0.77 (0.46–1.28) |
| ≧4 | 0.63 (0.29–1.35) | 0.15 (0.05–0.44) | 0.35 (0.19–0.65) |
| $x_1^2$ linear trend | 3.19 | 11.86[d] | 10.60[c] |
| $x_3^2$ heterogeneity | 5.67 | 18.31[d] | 12.73[b] |

[a]Based on observed numbers of subjects given in Table 5; [b]Significant at $P<0.01$; [c]Significant at $P<0.005$; [d]Significant at $P<0.001$.

In analyses comparing the observed birth order distribution with that which would be expected if the subjects were positioned at random within their sibships (Table V), teratoma patients were firstborn rather than later-born significantly more often than would be expected ($P<0.05$), but there were no other significant differences of observed from expected values; for seminoma and for all cases the observed/expected differences were in the same direction as the differences between the cases and the controls, but less strong and non-significant.

To investigate further the extent to which differences between the cases and controls were primarily in birth order or in sibship size, comparison was made between sibship size distribution of the cases and that expected on the basis of the sibship size within birth order distribution of the controls, and also between the observed birth order distribution of the cases and that expected on the basis of the birth order within sibship size distribution of the controls. The results of the comparison for sibship size are shown in Table VI; the results for birth order were similar to those in Table V, and all non-significant, and are not therefore presented. For teratoma, the difference from controls in these analyses was largely for birth order i.e. teratoma patients were first-born more often than expected; for seminoma and for all cases there were substantial differences from control-based expectations both for birth order and for sibship size i.e. these patients tended both to be of earlier birth order and to be from smaller sibships than would be expected, although the sibship size effect was larger, and was significant for seminoma ($x_4^2 = 12.46$, $P<0.02$).

**Table III** Risk of testis cancer according to sibship size

| | *Number (per cent)* | | | | *Relative risk* | | |
|---|---|---|---|---|---|---|---|
| *Number of sibs*[a] | *Teratoma* | *Seminoma* | *All cases* | *All controls* | *Teratoma* | *Seminoma* | *All cases* |
| 0 | 14 (14%) | 32 (23%) | 46 (18%) | 57 (12%) | 1.0 | 1.0 | 1.0 |
| 1 | 28 (27%) | 40 (29%) | 70 (27%) | 147 (30%) | 0.56 | 0.60 | 0.58 |
| 2 | 22 (21%) | 28 (20%) | 56 (22%) | 96 (20%) | 0.69 | 0.54 | 0.66 |
| 3 | 17 (17%) | 17 (12%) | 37 (14%) | 61 (12%) | 0.98 | 0.51 | 0.71 |
| ≧4 | 22 (21%) | 20 (15%) | 48 (19%) | 128 (26%) | 0.50 | 0.23 | 0.38 |
| Total | 103[b] (100%) | 137[b] (100%) | 257[b] (100%) | 489 (100%) | | | |
| | | | | $\chi_1^2$ linear trend | 0.54 | 16.17[e] | 7.99[d] |
| | | | | $\chi_4^2$ heterogeneity | 5.22 | 18.51[e] | 13.83[c] |

[a]Excluding stillbirths, and counting twins as two individuals; [b]1 teratoma and 1 seminoma patient are excluded because they were adopted and hence could give no information about sibs; [c]Significant at $P<0.01$; [d]Significant at $P<0.005$; [e]Significant at $P<0.001$.

**Table V** Birth order of subjects, and birth order expected on the basis of random allocation within their size of sibship

| | Teratoma | | Seminoma | | All cases | | All controls | |
|---|---|---|---|---|---|---|---|---|
| *Birth order*[a] | *Observed* | *Expected* | *Observed* | *Expected* | *Observed* | *Expected* | *Observed* | *Expected* |
| 1 | 55 (57%) | 43.7 (45%) | 70 (53%) | 68.6 (52%) | 129 (53%) | 116.8 (48%) | 201 (45%) | 189.7 (42%) |
| 2 | 21 (22%) | 27.7 (29%) | 40 (31%) | 35.6 (27%) | 66 (27%) | 67.8 (28%) | 125 (28%) | 134.7 (30%) |
| 3 | 10 (10%) | 13.7 (14%) | 17 (13%) | 15.1 (12%) | 31 (13%) | 31.3 (13%) | 59 (13%) | 60.2 (13%) |
| ≧4 | 11 (11%) | 12.0 (12%) | 4 (3%) | 11.6 (9%) | 16 (7%) | 26.0 (11%) | 64 (14%) | 64.5 (14%) |
| Total | 97 (100%) | 97.0 (100%) | 131 (100%) | 131.0 (100%) | 242 (100%) | 242.0 (100%) | 449 (100%) | 449.0 (100%) |
| $\chi_3^2$ | 5.61 | | 5.75 | | 5.18 | | 1.40 | |

None of the $x_3^2$ are significant.
[a]Including stillbirths and counting twins as one birth.

**Table VI** Sibship size of cases, and that expected on the basis of the sibship size by birth order distribution of controls

| | Teratoma | | Seminoma | | All cases | |
|---|---|---|---|---|---|---|
| *Sibship size*[b] | *Observed* | *Expected* | *Observed* | *Expected* | *Observed* | *Expected* |
| 0 | 16 | 15.0 | 33 | 19.2 | 49 | 35.3 |
| 1 | 28 | 33.8 | 41 | 50.0 | 73 | 88.1 |
| 2 | 21 | 18.5 | 27 | 28.8 | 53 | 51.1 |
| 3 | 14 | 12.0 | 13 | 16.4 | 28 | 30.3 |
| ≧4 | 18 | 17.6 | 17 | 16.6 | 39 | 37.2 |
| Total | 97 | 97.0 | 131 | 131.0 | 242 | 242.0 |
| $x_4^2$ | 1.71 | | 12.46[a] | | 8.25 | |

[a]$P<0.05$; [b]Including stillbirths, and counting twins as one birth.

As an indirect indicator of parental fertility for each subject the mean interval between births in the subject's sibship was calculated. The distribution of these intervals amongst cases, both overall and by histology, was very similar to that for controls.

*Age of parents at birth of the subject and at first-birth*

Although cases overall did not differ substantially from controls in the age of their mother at the subject's birth, for men born to nulliparae there was a significant gradient of increasing risk of testis cancer with increasing maternal age at their birth (Table VII); this gradient was steep and significant for seminoma but not teratoma. There was no such gradient of risk for men born to parous mothers. Risk of testis cancer in relation to mother's age at first-birth showed a highly significant gradient, again more marked for seminoma than teratoma, but this was due to the results for subjects born to nulliparae; there was no increase in risk with maternal age at first-birth for subjects who were not themselves firstborn. Results for paternal age at subject's birth and at first-birth approximately parallelled those for maternal age.

### Discussion

Some of the raised risks found in the study – for birth in general practitioner units, the sex ratio of sibs of teratoma patients, and several parental occupations and diseases – have not been studied previously and need re-examination in other data sets, but do not link to any obvious specific aetiological mechanism. For a few diseases in relatives there are published data, however. Tollerud *et al.* (1985) found cryptorchidism but not inguinal hernia to be more common in the first degree relatives of testicular cancer cases than of controls. Our data on cryptorchidism in relatives were weak because we deliberately did not ask directly about testicular conditions in order to avoid potential bias to the study. We did ask about herniae in relatives, and found a non-significant raised risk for seminoma. Tollerud *et al.* (1985) described a small number of testis cancer prone families within which inguinal hernia, hydrocoele and cryptorchidism were common, whilst Anderson *et al.* (1984) reported such a family in which cryptorchidism and dizygotic twinning were common. The case interviewed in our study whose brother had testis cancer came from a family with a variety of genitourinary defects. Also cases more often than controls in our study came from families in which more than one member had had hydrocoele, hernia or cryptorchidism (not solely as a direct corollary of the greater frequency of these conditions in cases themselves than in controls).

An excess of prostate conditions was reported in fathers of cases, but this may have been a reporting bias since operations for these are likely often to have happened at a similar time to the orchidectomies in the cases. An excess of lung cancer in parents was also noted, but this may not have been of aetiological importance since, as in a previous study (Henderson *et al.*, 1979), there was no excess of parents who were smokers. Some reported lung cancers in parents might have been lung metastases from primary tumours at other sites. The excesses of meningitis and of epulepsy in families of testis cancer patients follow non-significant excesses of cases with these diseases in the present study (Swerdlow *et al.*, 1987) and a significant excess of epulepsy in mothers of cases found elsewhere (Swerdlow *et al.*, 1982).

The relationships of testis cancer risk to handedness, sibship size, birth order, twinning and maternal age at delivery largely accord with the maternal hormone theory of testicular cancer aetiology, as will be discussed below, and thus give some new support to this theory. These results are not entirely as would be expected from the hormone hypothesis, however, and since none of the variables are directly hormonal, alternative explanations may apply.

**Table VII** Age of mother at birth of first child

| | Number (percent) | | | | Relative risk (95% CL) | | |
|---|---|---|---|---|---|---|---|
| | *Teratoma* | *Seminoma* | *All cases* | *All controls* | *Teratoma* | *Seminoma* | *All cases* |
| Age (years) | | | | | | | |
| *Firstborn subjects only* (= age of mother at subject's birth for men born to nulliparae) | | | | | | | |
| <20 | 3 (6%) | 3 (5%) | 6 (5%) | 24 (14%) | 1.0 | 1.0 | 1.0 |
| 20–24 | 16 (33%) | 14 (21%) | 32 (27%) | 56 (32%) | 2.6 (0.6–10.7) | 2.2 (0.5–9.4) | 2.6 (0.9–7.7) |
| 25–29 | 22 (46%) | 29 (44%) | 52 (44%) | 53 (30%) | 2.9 (0.7–11.6) | 3.3 (0.9–12.5) | 3.7 (1.3–10.3) |
| 30–34 | 4 (8%) | 9 (14%) | 14 (12%) | 34 (20%) | 1.0 (0.2–5.5) | 1.8 (0.4–8.6) | 1.5 (0.5–4.9) |
| 35–39 | 2 (4%) | 6 (9%) | 8 (7%) | 5 (3%) | 4.9 (0.5–43.7) | 13.9 (1.9–103.5) | 9.4 (1.9–46.4) |
| ≥40 | 1 (2%) | 5 (8%) | 6 (5%) | 2 (1%) | 2.5 (0.1–43.3) | 22.5 (1.3–383.7) | 11.6 (1.5–87.6) |
| Total | 48 (100%) | 66 (100%) | 118 (100%) | 174 (100%) | | | |
| | | | | $x_1^2$ linear trend | 0.10 | 6.55[b] | 4.57[a] |
| | | | | $x_5^2$ heterogeneity | 6.05 | 13.15[b] | 17.23[d] |
| *All subjects* | | | | | | | |
| <20 | 7 (9%) | 12 (10%) | 21 (10%) | 50 (13%) | 1.0 | 1.0 | 1.0 |
| 20–24 | 32 (39%) | 33 (27%) | 70 (32%) | 155 (39%) | 1.9 (0.7–4.8) | 1.0 (0.5–2.2) | 1.3 (0.7–2.4) |
| 25–29 | 32 (39%) | 53 (43%) | 87 (40%) | 124 (31%) | 1.8 (0.7–4.5) | 1.9 (1.3–2.6) | 1.8 (1.0–3.3) |
| 30–34 | 7 (9%) | 11 (9%) | 22 (10%) | 59 (15%) | 1.1 (0.3–3.4) | 0.8 (0.3–2.1) | 1.0 (0.5–2.1) |
| 35–39 | 3 (4%) | 8 (7%) | 11 (5%) | 8 (2%) | 4.7 (0.9–24.9) | 5.3 (1.5–19.0) | 4.3 (1.5–13.0) |
| ≥40 | 1 (1%) | 5 (4%) | 6 (3%) | 2 (1%) | 3.9 (0.3–52.7) | 14.5 (1.5–139.0) | 8.4 (1.4–49.8) |
| Total | 82 (100%) | 122 (100%) | 217 (100%) | 398 (100%) | | | |
| | | | | $x_1^2$ linear trend | 0.58 | 7.55[c] | 5.96[b] |
| | | | | $x_5^2$ heterogeneity | 5.55 | 21.41[e] | 16.92[d] |

[a]$P<0.05$; [b]$P<0.02$; [c]$P<0.01$; [d]$P<0.005$; [e]$P<0.001$.

The deficit of left-handed and ambidexterous men among men with testicular cancer, cryptorchidism and inguinal hernia might be related to maternal oestrogen levels in the following manner. High testosterone levels *in utero* may be a cause of left-handedness (Geschwind & Behan, 1982), and *in utero* excesses of oestrogen probably reduce testosterone secretion after mid-gestation by the foetal testis (Winter *et al.*, 1977). If, as has been hypothesised (Henderson *et al.*, 1979, 1983; Depue, 1984), *in utero* excesses of oestrogen cause genitourinary maldevelopment including effects on testicular development increasing the risk of later testis cancer, then it might be expected that the high oestrogen levels causing these conditions would also affect foetal testosterone secretion and hence handedness.

Firstborn men were at increased risk of testis cancer in the present data, and also in one smaller study (Depue *et al.*, 1983) but not another (Henderson *et al.*, 1979) nor a smaller study in children (Swerdlow *et al.*, 1982) although each had confidence limits compatible with the present findings. The maternal hormone hypothesis could explain a firstborn/later-born risk dichotomy (although it is not clear that it could explain a gradient of risk with birth order within the later-born, which appears to occur for seminoma). Available maternal oestrogen levels in the first trimester of pregnancy may be higher in mothers of firstborns (i.e. in nulliparous women) than in mothers of later born children, for two reasons. Firstly, nulliparous women have, when non-pregnant, higher plasma oestrogen levels, adjusted for cycle length, than do parous women (Bernstein *et al.*, 1985), and thus they might have higher levels also in early pregnancy. Secondly, levels of sex hormone binding globulin (SHBG), which binds oestrogens in a form unavailable to tissues, rise rapidly in early pregnancy (Uriel *et al.*, 1981) as oestrogen levels rise, and this increase in SHBG might proceed less rapidly during a woman's first experience of pregnancy than during her subsequent pregnancies (Depue *et al.*, 1983). Furthermore, there is evidence that SHBG levels are permanently increased following the first pregnancy (Bernstein *et al.*, 1985).

A raised risk of testis cancer in men from small sibships was found in the present work and also in the only other large published study (Morrison, 1976); the strength of the relationship by histology varied between these studies. Three smaller studies (Henderson *et al.*, 1979; Coldman *et al.*, 1982; Whittemore *et al.*, 1984) stated that they found no significant relation, but only one (Henderson *et al.*, 1979) presented data. Birth order is related to sibship size, and hence it would be expected that an aetiological relationship to one would be reflected in an association for the other. Thus testis cancer risk might be related to sibship size simply through a more direct relation of risk to birth order. For teratoma, comparison of the observed birth order with that expected from random allocation within sibships, and comparisons with the controls, suggested that this was the case. For seminoma, these comparisons suggested independent effects of both birth order and sibship size, although only the latter, which was stronger, gave significant results. The only previous study examining the risks by birth order allowing for sibship size (Morrison, 1976) stated that 'no substantial' effect was found, but did not present data.

To the extent that the sibship size associations of testis cancer are not explained by birth order, several possible mechanisms for an association of risk with sibship size can be postulated. Risk of testis cancer might be related to parental subfertility – for instance because of familial genito-urinary defects – and hence testis cancer cases might tend to come from small sibships; the analyses of inter-birth duration within the sibship were against this explanation, however, and in a previous study (Henderson *et al.*, 1979) risk of testis cancer was not raised for maternal difficulty in becoming pregnant. Testis cancer risk might be related to a particular childhood infection or to age at contracting the infection, as for instance occurs for risk of paralytic polio-myelitis, and sibship size might influence age-specific risks of the infection. Finally, sibship size might be related to testis cancer risk because of a non-causal association of sibship size with another, e.g. social class-related, aetiological factor for testis cancer.

Dizygotic twinning may be associated with high maternal gonadotrophin levels (Milham, 1964; Nylander, 1973). On the hormonal hypothesis one might therefore expect that testicular cancer would occur more often in men who were twins. The present data and those from a previous smaller study (Depue *et al.*, 1983) and a small study in children (Li & Fraumeni, 1972) were in this direction, but each was based on small numbers of twins: further data are needed. It is notable that congenital inguinal hernia (Chung & Myrianthopoulos, 1975; Czeizel, 1980; Depue, 1984), and in some studies cryptorchidism (Depue, 1984; Swerdlow *et al.*, unpublished), have also been found more common in twins than singleton-born subjects. The low birthweight of twins is an alternative possible reason why twinning might be associated with testis cancer and malformations of male genital tract development, for both of which there is some evidence of an association with low birthweight (Scorer & Farrington, 1971; Czeizel, 1980; Depue *et al.*, 1983; Depue, 1984).

The present study, like previous work (Henderson *et al.*, 1979; Coldman *et al.*, 1982; Swerdlow *et al.*, 1982), found no substantial relation in analyses of all subjects between age of parents at birth of the subject and risk of testis cancer. The risk has not previously been investigated by parity. The significant increase in risk of testis cancer, particularly seminoma, with increasing age of nulliparous mothers supports the maternal oestrogen theory of testis cancer aetiology. In one study plasma free oestradiol ($E_2$) levels in women have been found to increase with age, more steeply in nulliparous than in parous women (Bernstein *et al.*, 1985), with the highest level found in nulliparous older women. Overall plasma oestrone ($E_1$) and $E_2$ also rose with age, although the rise was not greater for nulliparous than parous women. Urinary oestrogens too have been shown to increase with age in nulliparous women (Trichopoulos *et al.*, 1980; Bernstein *et al.*, 1985) – in one study, the mean total follicular phase urinary oestrogen concentration in nulliparous 25–30 year old women was more than double that for nulliparous women age 17–19 years (Trichopoulos *et al.*, 1980). In parous women, a rise with age has been found less consistently; the present data showed no rise in testis cancer risk with maternal age for sons of parous women – further investigation is needed.

'Elderly primiparae' (primiparae aged over 35 years) are widely recognised to be at high risk of several medical complications of pregnancy and of abnormal labour (Willson *et al.*, 1983). Thus as an alternative to the maternal hormone hypothesis, raised risk of testis cancer in sons of elderly primiparae might be explained if one or more of the obstetric abnormalities occurring with increased frequency in such women were aetiological for testis cancer in the son.

If high maternal oestrogen levels are aetiological for testis cancer and if these high levels were to continue beyond the index pregnancy, then mothers of testis cancer patients might themselves be at high risk of cancers thought to be oestrogen-associated (i.e. breast and endometrial cancers (Henderson *et al.*, 1982)). The present data did not show such a raised risk, but the data were too few to examine this satisfactorily. A significant excess of seminoma patients did report a sister with breast cancer, however. Might increased maternal oestrogen levels in pregnancy affect risk of breast cancer in daughters as well as risk of testis cancer in sons? This would accord with previously unexplained reports that risk of breast cancer is raised for daughters of older women (Standfast, 1967; Henderson *et al.*, 1974; Rothman *et al.*, 1980).

The relation of testis cancer risk to age at delivery of nulliparous mothers in the present data was far more pronounced for seminoma than for teratoma, raising the possibility that high maternal oestrogen levels might particularly be a risk factor for seminoma. This would be of concern because the cohort of men exposed to diethylstilboestrol *in utero* are now reaching the age at which seminoma commonly occurs. There is some other evidence for a particular hormonal risk for seminoma, but very limited relevant data have been published by histology: cryptorchidism and congenital inguinal hernia are associated with greater risk of seminoma than non-seminoma histologies of testis cancer (Swerdlow *et al.*, 1987), as in the present data was being a twin. Risk of testis cancer in firstborn men was not, however, greater for seminoma than for teratoma in the present data. Further data on variables relevant to the maternal oestrogen hypothesis are needed by histology. Exploration is also needed of the oestrogen levels in mothers and the testis cancer risks in sons for combinations of potentially hormone-related risk factors: do overweight elderly primiparous mothers have especially high oestrogen levels, and do they give birth to sons at exceptionally high risk of testis cancer?

We thank the Cancer Research Campaign for funding the study; Professor Sir Richard Doll and Professor M.P. Vessey for advice, and Professor Vessey and Dr E.R. Rue for support; the clinicians, their staff and others, too numerous to thank individually here, who cooperated and helped with the study and provided notification of cases; Mrs M.A. Ainley and Miss M.M. Stone for their sensitive and skillful interviewing; Mrs F. Garven, Miss J. Clark, and Mr A. Radalowicz for computer programming; and Ms L. Semke for secretarial assistance.

### References

ANDERSON, K.C., LI, F.P. & MARCHETTO, D.J. (1984). Dizygotic twinning, cryptorchism, and seminoma in a sibship. *Cancer*, **53**, 374.

BERNSTEIN, L., PIKE, M.C., ROSS, R.K., JUDD, H.L., BROWN, J.B. & HENDERSON, B.E. (1985). Estrogen and sex hormone-binding globulin levels in nulliparous and parous women. *J. Natl Cancer Inst.*, **74**, 741.

BRESLOW, N.E. & DAY, N.E. (1980). Statistical methods in cancer research. **Vol. 1**—The Analysis of case-control studies. *International Agency for Research on Cancer, IARC Scientific Publications No. 32*, Lyon.

CHUNG, C.S. & MYRIANTHOPOULOS, N.C. (1975). Factors affecting risks of congenital malformations. 1. Epidemiologic analysis. In *Birth Defects*, Bergsma (ed) **Vol. XI**, No. 10. Stratton: New York.

COLDMAN, A.J., ELWOOD, J.M. & GALLAGHER, R.P. (1982). Sports activities and risk of testicular cancer. *Br. J. Cancer*, **46**, 749.

CZEIZEL, A. (1980). Epidemiologic characteristics of congenital inguinal hernia. *Helv. Paediat. Acta*, **35**, 57.

DEPUE, R.H. (1984). Maternal and gestational factors affecting the risk of cryptorchidism and inguinal hernia. *Int. J. Epidemiol.*, **13**, 311.

DEPUE, R.H., PIKE, M.C. & HENDERSON, B.E. (1983). Estrogen exposure during gestation and risk of testicular cancer. *J. Natl Cancer Inst.*, **71**, 1151.

GESCHWIND, N. & BEHAN, P. (1982). Left-handedness: association with immune disease, migraine, and developmental learning disorder. *Proc. Natl Acad. Sci. USA*, **79**, 5097.

HENDERSON, B.E., POWELL, D., ROSARIO, I. & 6 others (1974). An epidemiologic study of breast cancer. *J. Natl Cancer Inst.*, **53**, 609.

HENDERSON, B.E., BENTON, B., JING, J., YU, M.C. & PIKE, M.C. (1979). Risk factors for cancer of the testis in young men. *Int. J. Cancer*, **23**, 598.

HENDERSON, B.E., ROSS, R.K., PIKE, M.C. & CASAGRANDE, J.T. (1982). Endogenous hormones as a major factor in human cancer. *Cancer Res.*, **42**, 3232.

HENDERSON, B.E., ROSS, R.K., PIKE, M.C. & DEPUE, R.H. (1983). Epidemiology of testis cancer. In *Urological Cancer*, Skinner (ed), p. 237. Grune and Stratton: New York.

LI, F.P. & FRAUMENI, J.F. Jr. (1972). Testicular cancers in children: epidemiologic characteristics. *J. Natl Cancer Inst.*, **48,** 1575.

LOUGHLIN, J.E., ROBBOY, S.J. & MORRISON, A.S. (1980). Risk factors for cancer of the testis. *N. Engl. J. Med.*, **303,** 112.

MILHAM, S. Jr. (1964). Pituitary gonadotrophin and dizygotic twinning. *Lancet*, **ii,** 566.

MORRISON, A.S. (1976). Some social and medical characteristics of army men with testicular cancer. *Am. J. Epidemiol.*, **104,** 511.

NYLANDER, P.P.S. (1973). Serum levels of gonadotrophins in relation to multiple pregnancy in Nigeria. *J. Obs. Gynaec. Brit. Commonwealth*, **80,** 651.

OFFICE OF POPULATION CENSUSES AND SURVEYS. (1970). *Classification of Occupations* 1970, HMSO: London.

OFFICE OF POPULATION CENSUSES AND SURVEYS. (1982). *Birth Statistics*. England and Wales, 1980. Series FM1 No. 7. HMSO: London.

REGISTRAR GENERAL. (1949). *Statistical Review of England and Wales for the year 1947*. Tables, Part II. Civil. HMSO: London.

ROTHMAN, K.J., MacMAHON, B., LIN, T.M. & 6 others (1980). Maternal age and birth rank of women with breast cancer. *J. Natl Cancer Inst.*, **65,** 719.

SCHOTTENFELD, D., WARSHAUER, M.E., SHERLOCK, S., ZAUBER, A.G., LEDER, M. & PAYNE, R. (1980). The epidemiology of testicular cancer in young adults. *Am. J. Epidemiol.*, **112,** 232.

SCORER, C.G. & FARRINGTON, G.H. (1971). *Congenital deformities of the testis and epididymis*. Butterworths: London.

STANDFAST, S.J. (1967). Birth characteristics of women dying from breast cancer. *J. Natl Cancer Inst.*, **39,** 33.

STORER, B.E., WACHOLDER, S. & BRESLOW, N.E. (1983). Maximum likelihood fitting of general risk models to stratified data. *Appl. Statistics*, **32,** 172.

SWERDLOW, A.J., STILLER, C.A. & KINNIER WILSON, L.M. (1982). Prenatal factors in the aetiology of testicular cancer: an epidemiological study of childhood testicular cancer deaths in Great Britain, 1953–73. *J. Epidemiol. Commun. Hlth*, **36,** 96.

SWERDLOW, A.J., HUTTLY, S.R.A. & SMITH, P.G. (1987). Testicular cancer and antecedent diseases. *Br. J. Cancer*, **55,** 97–103.

TOLLERUD, D.J., BLATTNER, W.A., FRASER, M.C. & 9 others (1985). Familial testicular cancer and urogenital developmental anomalies. *Cancer*, **55,** 1849.

TRICHOPOULOS, D., COLE, P., BROWN, J.B., GOLDMAN, M.B. & MacMAHON, B. (1980). Estrogen profiles of primiparous and nulliparous women in Athens, Greece. *J. Natl Cancer Inst.*, **65,** 43.

URIEL, J., DUPIERS, M., RIMBAUT, C. & BUFFE, D. (1981). Maternal serum levels of sex steroid-binding protein during pregnancy. *Br. J. Obstet. Gynaecol.*, **88,** 1229.

WHITTEMORE, A.S., PAFFENBARGER, R.S. Jr., ANDERSON, K. & LEE, J.E. (1984). Early precursors of urogenital cancers in former college men. *J. Urol.*, **132,** 1256.

WILLSON, J.R., CARRINGTON, E.R. & LEDGER, W.J. (1983). *Obstetrics and Gynecology*. C.V. Mosby: St. Louis.

WINTER, J.S.D., FAIMAN, C. & REYES, F.I. (1977). Sex steroid production by the human fetus: its role in morphogenesis and control by gonadotropins. In *Morphogenesis and Malformation of the Genital System*, Blandau & Bergsma (eds), Birth Defects, **Vol. XIII,** No. 2, p. 41. Alan R. Liss: New York.

WORLD HEALTH ORGANIZATION (1978) *International Classification of Diseases*. Ninth Revision. **Vol. 1.** WHO: Geneva.